# CXCR4 expression in lung carcinogenesis: Evaluating gender-specific differences in survival outcomes based on CXCR4 expression in early stage non-small cell lung cancer patients

**DOI:** 10.1371/journal.pone.0241240

**Published:** 2021-01-28

**Authors:** Andrea S. Fung, Karen Kopciuk, Michelle L. Dean, Adrijana D’Silva, Shannon Otsuka, Alexander Klimowicz, Desiree Hao, Don Morris, D. Gwyn Bebb

**Affiliations:** 1 Tom Baker Cancer Centre, Calgary, Alberta, Canada; 2 Department of Oncology, Cumming School of Medicine, University of Calgary, Calgary, Alberta, Canada; 3 Department of Oncology, Queen’s University, Kingston, Ontario, Canada; Baylor College of Medicine, UNITED STATES

## Abstract

**Introduction:**

Evidence suggests that the expression of certain cytokine receptors increases with lung cancer evolution. Overexpression of the cytokine receptor CXCR4 is associated with poor outcomes in stage IV non-small cell lung cancer (NSCLC), with shorter survival in females with high CXCR4 expression. This study quantifies CXCR4 expression in early stage disease and evaluates its association with gender-specific recurrence-free (RFS) and overall survival (OS) in resected stage I-III NSCLC patients.

**Methods:**

Patient characteristics and clinical outcomes were obtained from the Glans-Look Lung Cancer (G-LLC) database for early stage NSCLC patients diagnosed between 2003–2006 at the Tom Baker Cancer Centre (TBCC). CXCR4 expression was quantified on tissue microarrays (TMA). Median RFS and OS were evaluated by gender using Kaplan-Meier analyses. CXCR4 expression and outcome data were analyzed using Cox proportional hazards (PH) and multi-state models (MSM).

**Results:**

176 stage I-III NSCLC patients were identified. CXCR4 expression was lower in early stage NSCLC patients, with a mean CXCR4 expression of 1729 (SD 1083) compared to 2640 (SD 1541) in stage IV patients. On Kaplan-Meier, median RFS by gender was similar (male 52.8 months vs. female 54.5 months) as was median OS (male 80.9 months vs. female 89.0 months), and there was no significant difference in RFS (p = 0.60) or OS (p = 0.30) by gender and CXCR4 groups over follow-up. By multivariable analysis, CXCR4 expression was not prognostic for RFS (Hazard Ratio (HR) = 1.00, p = 0.73) or OS (HR = 1.00, p = 0.44), and no gender difference was observed.

**Conclusions:**

CXCR4 expression increases with stage progression in NSCLC but is not prognostic in early stage NSCLC patients of either gender. Mechanisms by which CXCR4 expression increases during lung carcinogenesis warrant further exploration and testing in clinical trials.

## Introduction

Cytokines and their receptors have been implicated in tumor progression [1–3], and evidence suggests that the expression of some cytokine receptors increases with cancer evolution [4]. The cytokine receptor CXCR4 and its ligand stromal cell derived factor-1 (SDF-1) can activate signaling through the phosphoinositol-3-kinase (PI3K) and mitogen activated protein kinase (MAPK) pathways, and have been implicated in cell migration, invasion and angiogenesis [5–8]. Furthermore, the CXCR4/SDF-1 axis may be involved in regulating development of organ-specific metastases [5].

Overexpression of CXCR4 has been associated with poor outcomes in patients with advanced cancer, including breast, head and neck, ovarian, and non-small cell lung cancer (NSCLC), among others [4, 9–14]. Increased CXCR4 expression has been associated with nodal and distant metastases, and worse survival in NSCLC patients [7, 8, 15, 16]. Studies suggest that nuclear or cytomembranous CXCR4 expression is differentially associated with survival, with better survival outcomes associated with nuclear staining, and an association between distant metastases and worse survival with cytomembranous staining [8, 16, 17].

Previously, we demonstrated a gender-specific difference in outcomes of stage IV NSCLC patients according to CXCR4 expression, with shorter survival in females with high CXCR4 expression [14]. However, the extent of CXCR4 expression in early versus advanced NSCLC and its relationship to outcome and gender is not known. Here, we aimed to characterize gender-specific differences in survival based on CXCR4 expression in resected stage I-III NSCLC patients.

## Materials and methods

### Patient characteristics

A retrospective analysis of stage I-III NSCLC patients (AJCC 7^th^ edition) diagnosed at the Tom Baker Cancer Centre (Calgary, Canada) between 2003–2006 was undertaken. Patient characteristics, diagnostic, treatment and outcome data were collected through chart review and kept in the Glans-Look Lung Cancer (G-LLC) database. Stage IV patient data were also collected from the G-LLC database. All patient data and tissue samples were de-identified, and patient records and tissue samples were accessed for analyses from August 2009 to July 2010. Research ethics board (REB) approval was obtained from the Health Research Ethics Board of Alberta Cancer Committee (HREBA-CC) prior to study initiation, and research was completed in accordance with the Tri-Council Policy Statement on Research with Human Subjects.

### Quantification of CXCR4 expression

Tissue microarrays (TMA) were generated from formalin-fixed paraffin-embedded samples from archived biopsy or surgical specimens. Representative cores (0.6mm) were taken in triplicate from each specimen. Fluorescent immunohistochemical staining (IHC), quantification and analysis of CXCR4 expression were completed as previously described by Otsuka *et al*. [14]. Briefly, 5 μm sections were cut from TMA blocks and stained using a 1:500 dilution of anti-pan-cytokeratin mouse monoclonal antibody (Dako) and a 1:25 dilution of an anti-CXCR4 rabbit monoclonal antibody (clone UMB2, Biotrend, Köln, Germany) to identify tumor cells and CXCR4 expression, respectively [14]. Images were acquired using filters for DAPI (nuclear compartment), Cy3 (cytokeratin-positive tumor cells and the cytosolic compartment), and Cy5 (CXCR4 biomarker). Nuclear and cytomembranous CXCR4 expression was characterized and CXCR4 levels were expressed as a tumor-specific automated quantitative analysis (AQUA) score, which represented the maximum signal intensity per tumor area [14]. A definite AQUA score cut-point to differentiate between low vs. high CXCR4 expression was not identified; therefore, mean CXCR4_max_ expression, in addition to ranges of AQUA scores were utilized to compare the level of CXCR4 expression between groups.

### Statistical analysis

Survival outcomes included recurrence, recurrence-free survival (RFS), defined as the time from the end of treatment to recurrence or death; and overall survival (OS), defined as time from diagnosis to death from any cause. CXCR4 expression and survival outcomes were analyzed using Kaplan-Meier curves, Cox proportional hazards (PH) and multi-state models (MSM) [18], which accounted for the effect of treatments received following recurrence. CXCR4 expression levels were compared between groups using a two sample *t*-test. Statistical analyses were completed using R version 3.5.1 software.

## Results

### Patient characteristics

One hundred and seventy six stage I-III patients with TMA-derived CXCR4 data were identified. Baseline patient characteristics are summarized in Table 1. The mean age at diagnosis was 65. There were 52.8% females, with 25.6% current smokers, 60.8% ex-smokers and 11.9% never smokers. There were 114 patients (64.8%) with adenocarcinoma, 59.7% had stage I disease, and 77.3% were lymph node negative (N0). Over a maximum follow up of 17.4 years, there were 128 deaths (72.7%) and 88 patients (50%) had a recurrence (Table 1).

**Table 1:** Frequency and percent of patient and tumor characteristics at diagnosis for all patients and by gender.

	Stage I-III	%	Female (n = 93)	%	Male	%	Stage IV	%
	(n = 176)				(n = 83)		(n = 147)	
**Gender**								
Female	93	52.8	93	100	-	-	75	51.0
Male	83	47.2	-	-	83	100	72	49.0
**Smoking Status**								
Current	45	25.6	25	26.9	20	24.1	44	30.0
Ex-smoker	107	60.8	48	51.6	59	71.1	78	53.1
Never	21	11.9	19	20.4	2	2.4	20	13.6
Unknown	3	1.7	1	1.1	2	2.4	5	3.4
**Stage**								
IA	39	22.2	25	26.9	14	16.9		
IB	66	37.5	36	38.7	30	36.2		
IIA	26	14.8	9	9.7	17	20.5		
IIB	28	15.9	14	15.1	14	16.9		
IIIA	17	9.7	9	9.7	8	9.6		
IV							147	100
**Histology**								
Adenocarcinoma	114	64.8	70	75.3	44	53	74	50.3
Squamous cell carcinoma	51	29	14	15.1	37	44.6	38	25.9
Adenosquamous carcinoma	3	1.7	2	2.2	1	1.2	1	0.7
Large cell carcinoma	7	4	6	6.5	1	1.2	10	6.8
Other NSCLC	1	0.6	1	1.1	0	0	24	16.3
**Nodal status**								
N0	136	77.3	73	78.5	63	75.9	21	14.3
N1	31	17.6	16	17.2	15	18.1	6	4.1
N2	9	5.1	4	4.3	5	6	77	52.4
N3							24	16.3
X							19	12.9
**Chemotherapy**								
Yes	75	42.6	37	39.8	38	45.8		
- Neoadjuvant/Adjuvant	62	35.2	31	33.3	31	37.4		
- Palliative	13	7.4	6	6.5	7	8.4	36	24.5
No	101	57.4	56	60.2	45	54.2	111	75.5
**Radiation therapy**								
Yes	60	34.1	31	33.3	29	34.9		
- Neoadjuvant/Adjuvant	18	10.2	10	10.8	8	9.6		
- Palliative	42	23.9	21	22.6	21	25.3	118	80.3
No	116	65.9	62	66.7	54	65.1	29	19.7
**CXCR4 (max)**								
Mean (25%, 75%)	1729	(1096, 1937)	1567	(1050, 1712)	1911	(1174, 2237)	2640	(1,681, 3,151)
**Age at Diagnosis**								
Mean (25%, 75%)	65	(58.3, 73.4)	64.7	(58.0, 73.4)	65.5	(59.3, 73.2)	67.6	(59.5, 76.0)
**Recurrence**								
Yes	88	50	49	52.7	39	47		
No	88	50	44	47.3	44	53		
**Deaths**								
Yes	128	72.7	65	69.9	63	75.9	147	100
No	48	27.3	28	30.1	20	24.1	0	

An updated analysis of 147 stage IV patients (75 females, 72 males) identified in the G-LLC database was also performed with a detailed summary of their characteristics also shown in Table 1.

Of all stage I-III patients, 62 patients (35.2%) received neoadjuvant/adjuvant chemotherapy, while 18 (10.2%) received adjuvant radiation. Of the 88 patients who recurred, 29 (33.0%) received systemic treatment (chemotherapy, targeted therapy or immunotherapy) post-recurrence, and 54 (61.4%) received radiation (Table 2). The treatments received after recurrence was similar across genders.

**Table 2:** Treatments received after recurrence for all patients and by gender.

At Recurrence	All (n = 88)	%	Female (n = 49)	%	Male (n = 39)	%
**Any treatment post recurrence**						
Yes	62	70.5	34	69.4	28	71.8
No	26	29.5	15	30.6	11	28.2
**Systemic treatment post recurrence**						
Yes	29	33.0	16	32.7	13	33.3
No	59	67.1	33	67.4	26	66.7
**Radiation post recurrence**						
Yes	54	61.4	30	61.2	24	61.5
No	34	38.6	19	38.8	15	38.5

### CXCR4 expression

**Fig 1:**
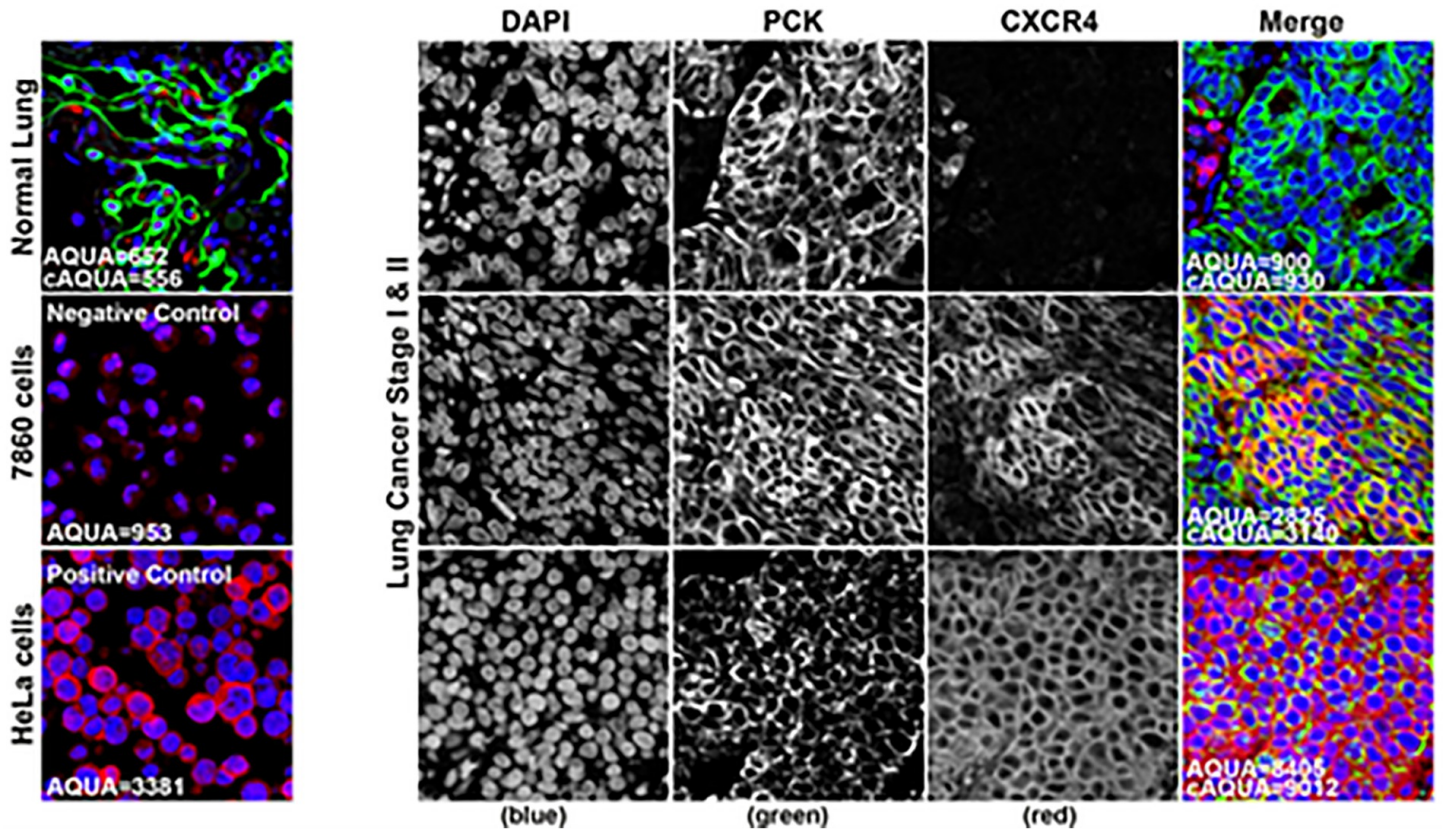
CXCR4 immunohistochemical (IHC) staining. CXCR4 expression in negative and positive (HeLa) control cells (left panel). CXCR4 staining in early stage non-small cell lung cancer patients, with low (right top panel) and high CXCR4 expression (right bottom panel) shown in red. DAPI nuclear staining shown in blue, and PCK tumor cytosolic component shown in green.

**Fig 2:**
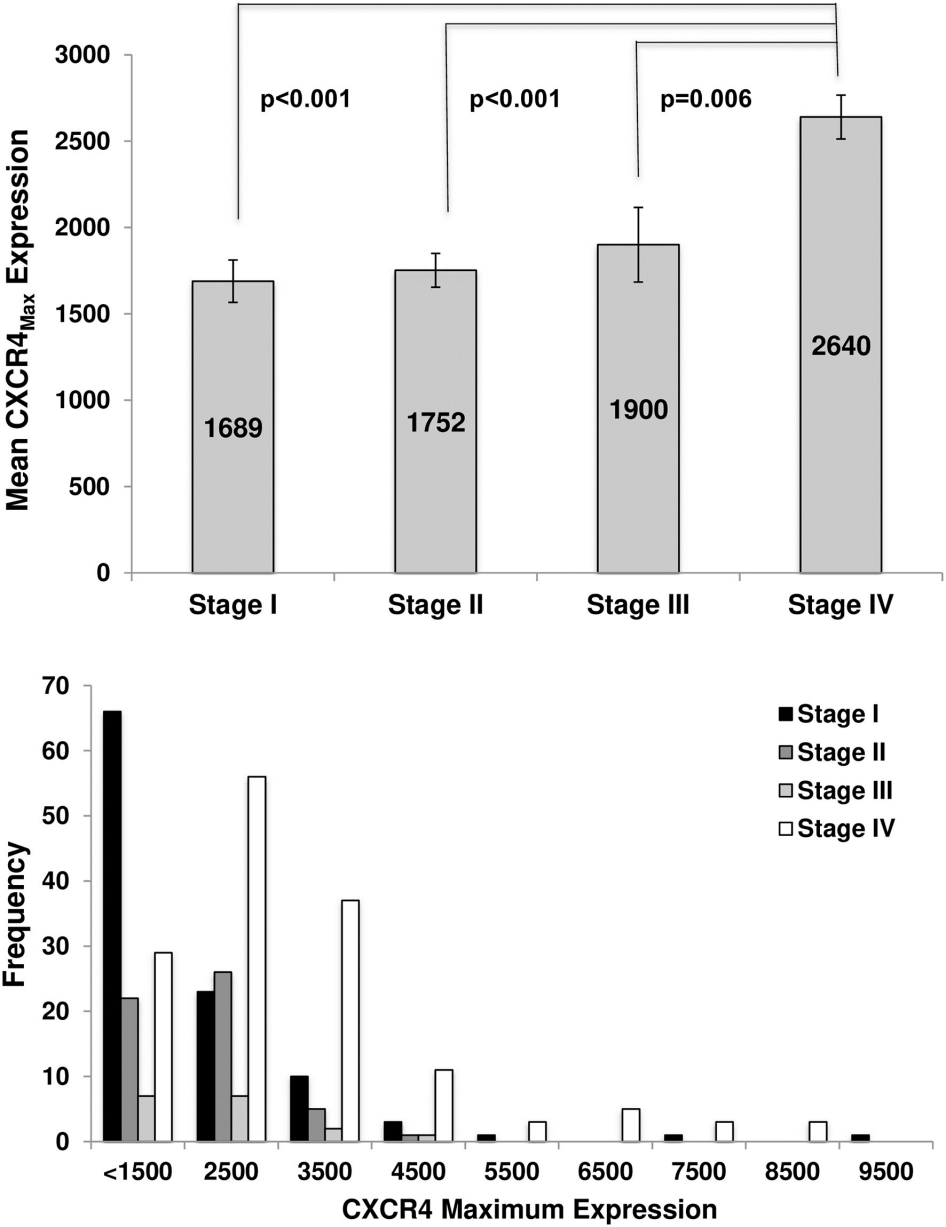
CXCR4 expression is lower in early stage compared to late stage NSCLC patients. Mean CXCR4_Max_ expression increased with stage progression, with higher CXCR4 expression in stage IV NSCLC patients (top panel); error bars represent standard error of the mean. Maximum CXCR4 expression levels >2500 were less frequent in early stage lung cancer patients compared to late stage patients (bottom panel).

A range of CXCR4 expression was observed in early stage NSCLC patients; representative IHC images showing low versus high CXCR4 expression are shown in Fig 1. Mean CXCR4_Max_ expression was 1729 (SD 1083) in stage I-III patients, which was lower than the mean of 2640 (SD 1541) observed in stage IV patients. There appeared to be an increase in mean CXCR4_Max_ expression with stage progression, with a statistically significant difference in mean CXCR4_Max_ expression between stage I vs. IV (1689 ±1257 vs. 2640 ±1541, p<0.001), stage II vs. IV (1752 ±719 vs. 2640 ±1541, p<0.001) and stage III vs. IV (1900 ±890 vs. 2640 ±1541, p = 0.006) patients (Fig 2 top panel); however, the differences in CXCR4 expression between stage I vs. II, I vs. III, and II vs. III patients were not statistically significant (p-values >0.05). Maximum CXCR4 expression levels >2500 were more frequent in stage IV versus stage I-III patients (Fig 2 bottom panel).

The nuclear and cytomembranous components of CXCR4 staining were both higher in stage IV patients compared to early stage patients (p<0.05), and this was consistent across genders. Interestingly, males with early stage NSCLC had higher nuclear expression of CXCR4 than females (mean of 1911 ±1107 vs. 1567 ±1040, p = 0.049). Likewise, nuclear CXCR4_Max_ expression levels >2500 were more frequent in males compared to females (18.1% vs. 10.7%) in early stage NSCLC patients.

**Fig 3:**
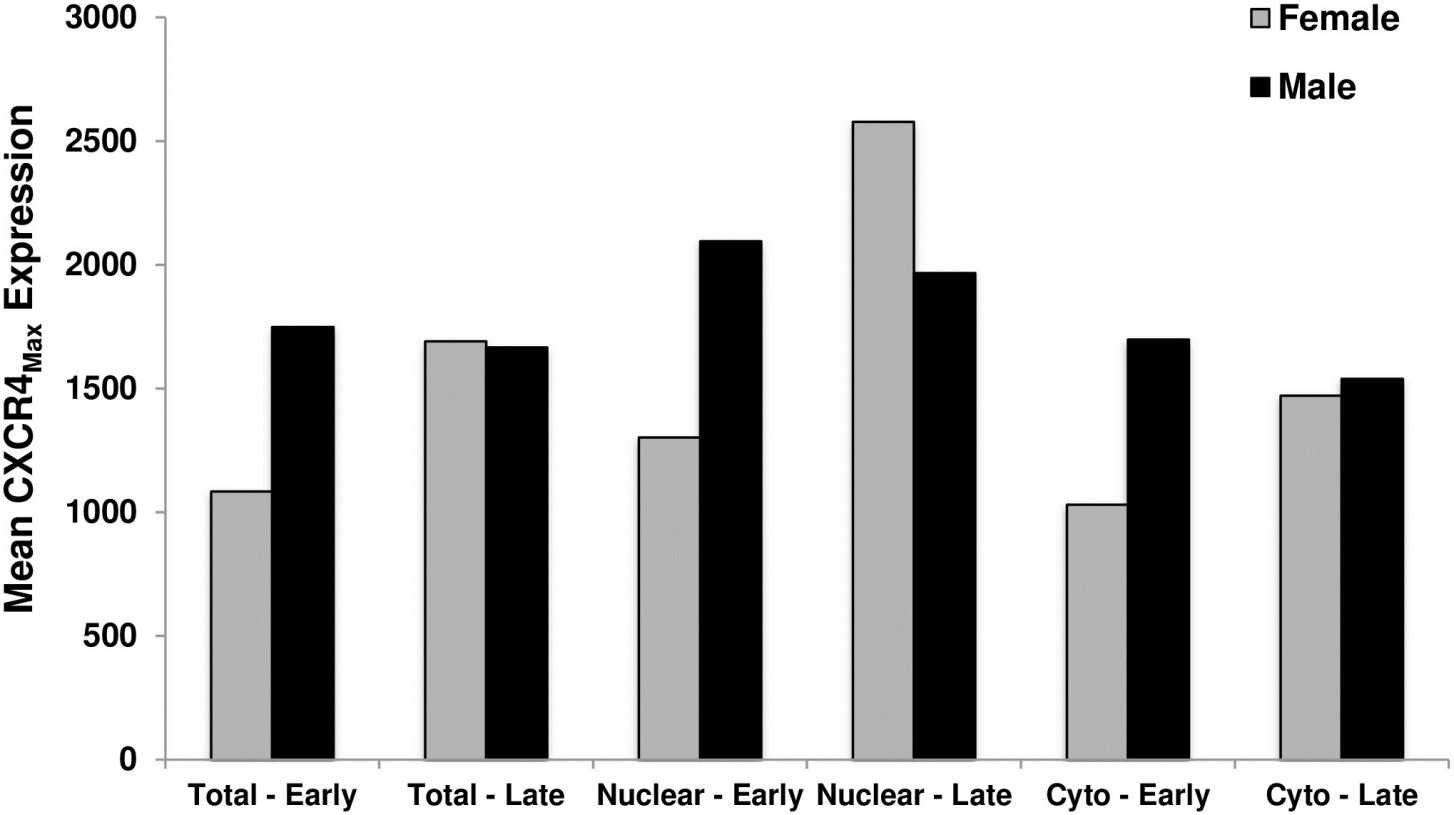
Change in nuclear and cytomembranous CXCR4 expression in two patients with both early and late stage tissue samples. There was an increase in both the nuclear and cytomembranous (cyto) CXCR4 expression in the late stage (stage IV) sample compared to the early stage sample in the female patient (grey bars). Conversely, there was no difference in CXCR4 expression in the male patient, irrespective of stage (black bars).

Two patients had both resected and recurrent metastatic tissue available—the female had higher CXCR4 expression (both nuclear and cytomembranous) in the stage IV than in the early stage sample, whereas the male patient’s CXCR4 expression was similar, irrespective of stage (Fig 3). Both patients recurred, with the male recurring locally and the female developing distant metastatic recurrence.

### Recurrence and survival outcomes

**Fig 4:**
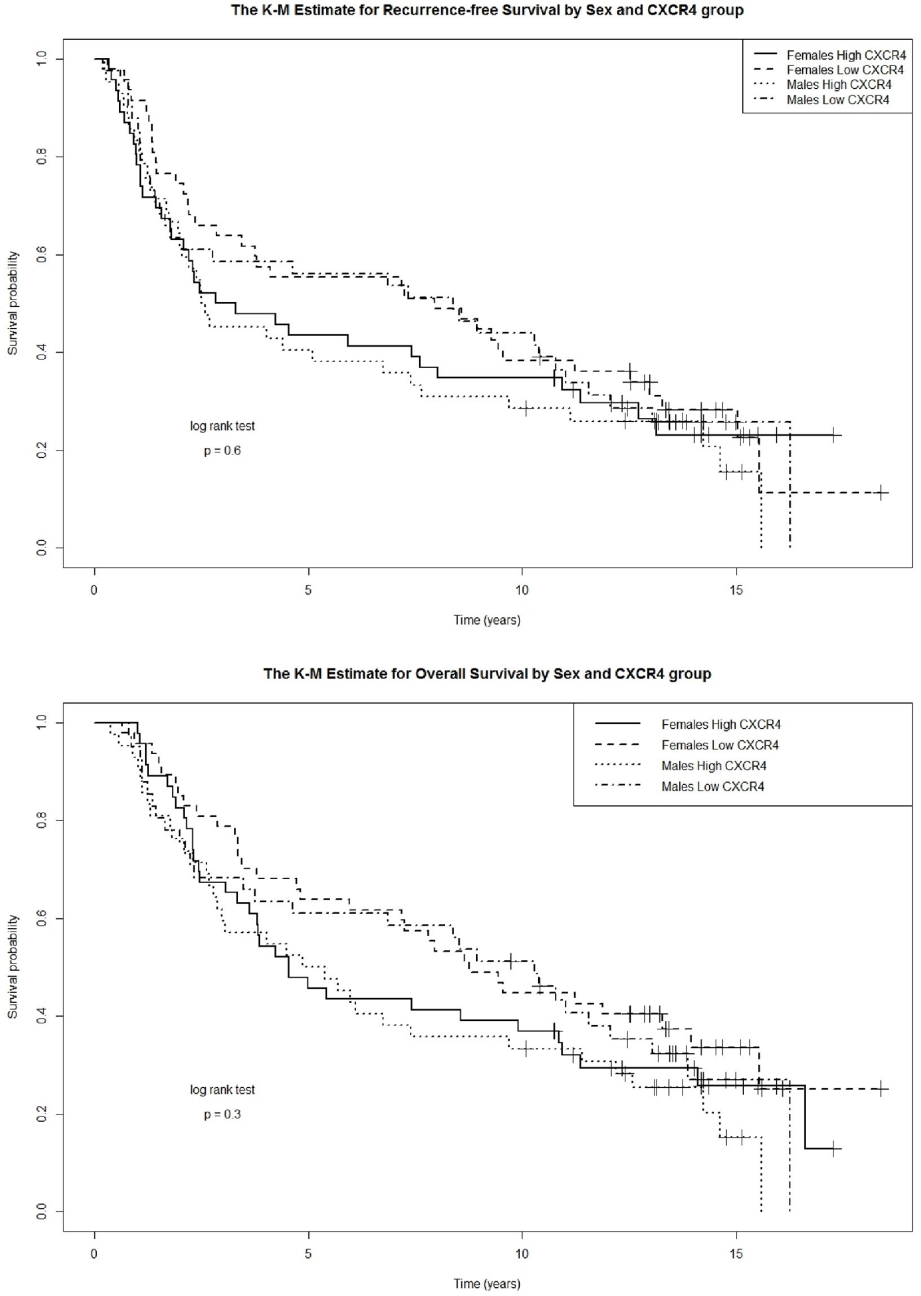
No gender-specific difference in survival outcomes by CXCR4 expression. There was no difference in recurrence-free survival (top panel) or overall survival (bottom panel) based on gender in patients with high or low levels of CXCR4 expression based on median splits.

In this early stage patient cohort, CXCR4 expression did not independently predict for recurrence, RFS or OS (Table 3). Similarly, there was no differential predictive effect by gender or following recurrence for death in the multi-state model (Table 3). On Kaplan-Meier analysis, no gender difference in RFS (p = 0.60) or OS (p = 0.30) by gender and CXCR4 expression groups based on median splits were observed and median survival times were similar (RFS: male 52.8 months vs. female 54.5 months, OS: male 80.9 months vs. female 89.0 months; Fig 4 top and bottom panels, respectively). However, patients of either gender with CXCR4_Max_ expression greater than the median split appeared to have a trend towards worse RFS and OS starting approximately 3 years from diagnosis. When stage IV patients were included with the early stage patients in a multivariable adjusted Cox PH model, CXCR4 expression did not independently, or synergistically with gender or stage, predict for OS (HR = 1.0001, p = 0.32).

**Table 3:** Impact of CXCR4 expression on recurrence, RFS and OS.

Recurrence	HR	95% CI	Pr(>|z|)
CXCR4 (max)	1.0000	0.9998, 1.0000	0.87
**RFS**			
CXCR4 (max)	1.0000	0.9998, 1.0000	0.73
**OS**			
CXCR4 (max)	1.0001	0.9999, 1.0000	0.44
**Multi-state** (recurrence—death transition)			
CXCR4 (max)	1.0002	0.9999, 1.0004	0.08

*All Cox PH models adjusted for stage, nodal status, histology, gender, age at diagnosis, smoking status, systemic and radiation therapy before recurrence; the extended stay Markov PH multistate model included post recurrence treatment

## Discussion

In this study, we report that CXCR4 expression in early stage resected NSCLC patients was substantially lower than previously reported for stage IV patients by Otsuka *et al*. [14]. Our data showed an increase in CXCR4 expression with stage progression. We observed an increase in both the nuclear and cytomembranous components of CXCR4 expression in late stage patients when compared to early stage patients. In contrast to metastatic NSCLC, we found that CXCR4 expression was not prognostic of survival outcomes in stage I-III patients. However, there was a trend towards worse RFS and OS in patients with a CXCR4_Max_ expression greater than the median split in either gender. Overall, our data suggest that CXCR4 expression increases with cancer progression, acquiring clinical significance only in the metastatic setting. A role for chemokine receptor axes in tumor progression, invasion and metastasis has been suggested before [1–4, 6]. Our analysis supports an association between tumor progression and CXCR4 expression in NSCLC, but does not address causality.

Our cohort included a small number of patients with stage IIIA disease, no stage IIIB patients, and only 9 patients with N2 disease. Consequently, analysis of the impact of CXCR4 expression on survival outcomes in stage III patients was limited. On multivariable survival analysis, we found no association between CXCR4 expression and lymph node status in early stage patients; however, our study was limited by a small number of node-positive patients and a robust analysis could not be completed. A plausible explanation is that the resected stage III cases represent those with minimal presurgical evidence of nodal disease based on imaging and mediastinal staging. In such cases, the extent of metastatic capability is likely not as well developed as in cases where disease has metastasized to distal organs.

A meta-analysis by Liang *et al*. found an association between CXCR4 overexpression and lymph node metastasis, distant metastasis, and OS [15]; however, the patient population was quite heterogeneous (i.e. included stage I-IV patients as well as small cell lung cancer patients), making comparison to our patient population difficult. It is possible that the impact of the CXCR4/SDF-1 axis is more prominent in patients with lymph node metastasis, or after recurrence, as this pathway is implicated in cell migration, invasion, and possible homing to different organ sites of metastases [4, 7, 8, 19]. Interestingly, the majority of stage IV patients (n = 107, 72.8%) in our study had node-positive disease, suggesting a possible relationship between higher CXCR4 levels in stage IV patients and development of lymph node and distant metastases. Further studies should be considered to evaluate the association between CXCR4 expression (nuclear vs. cytomembranous) and development of organ-specific sites of metastases.

Otsuka *et al*. previously reported a gender-specific difference in survival, with worse outcomes in females with high CXCR4 expression [14]. We did not observe a difference in survival outcomes based on gender; however, we did note higher nuclear CXCR4 expression in males compared to females in the early stage setting. Previous studies have shown that nuclear expression of CXCR4 is associated with better survival than cytomembranous expression, and this might be due to less tumor-promoting effects of the receptor with nuclear localization compared to when the receptor is in the cytomembranous compartment [8, 16]. It is possible that the greater nuclear expression noted in males with early stage NSCLC could account for the different survival outcomes between genders. In addition, differences in nuclear vs. cytomembranous expression could lead to different risks for development of recurrent or metastatic disease.

There are limitations in the current study. Firstly, we are limited by the retrospective nature of the analysis; however, our data collection was comprehensive and included a substantial number of early stage patients in comparison to other published studies evaluating CXCR4 expression in lung cancer patients. Secondly, the small number of stage III patients (with no stage IIIB patients), as well as few node-positive patients in our population, did not allow for a robust analysis of more advanced stage III disease. However, despite this, we were still able to show an increase in CXCR4 expression with stage progression. Finally, there was limited data on sites of metastases in patients who recurred; therefore, we were unable to evaluate for an association between CXCR4 expression and development of organ-specific sites of metastases.

Targeted therapies have been developed to inhibit the CXCR4/SDF-1 axis, and have been evaluated in various cancers [2, 20–22]. Despite disappointing results in trials utilizing anti-CXCR4 strategies [23, 24], the CXCR4/SDF-1 axis warrants further study to define its role in tumor progression, recurrence, and metastasis, as well as elucidate the impact on the tumor microenvironment. Furthermore, the function of the CXCR4/SDF-1 axis in immune biology suggests a potential therapeutic role for anti-CXCR4 strategies with immunotherapy [25]. Trials are ongoing to evaluate this combination (NCT03193190, NCT03281369, NCT03337698).

## Conclusion

CXCR4 expression is significantly lower in stage I-III than in stage IV NSCLC patients. Its expression does not predict survival outcomes in early stage patients, and no gender difference was identified between CXCR4 expression and survival. Mechanisms by which CXCR4 expression increases during lung carcinogenesis warrant further exploration and testing in clinical trials.
